# ASO Author Reflections: Personalized Breast Cancer Treatment Using Preoperative Partial Breast Irradiation

**DOI:** 10.1245/s10434-023-13298-6

**Published:** 2023-03-09

**Authors:** Yasmin A. Civil, Lysanne W. Jonker, Arlene L. Oei, Susanne van der Velde, H. J. G. Desirée van den Bongard

**Affiliations:** 1 Department of Radiation Oncology, Amsterdam UMC Location Vrije Universiteit Amsterdam, Amsterdam, The Netherlands; 2 Cancer Center Amsterdam, Cancer Treatment and Quality of Life, Amsterdam, The Netherlands; 3 Laboratory for Experimental Oncology and Radiobiology, Amsterdam UMC Location University of Amsterdam, Amsterdam, The Netherlands; 4 Center for Experimental Molecular Medicine, Amsterdam UMC Location University of Amsterdam, Amsterdam, The Netherlands; 5 Cancer Center Amsterdam, Cancer Biology and Immunology, Amsterdam, The Netherlands; 6 Department of Surgery, Amsterdam UMC Location Vrije Universiteit Amsterdam, Amsterdam, The Netherlands

## Past

Standard of care for low-risk breast cancer patients is breast conserving surgery (BCS) followed by partial breast irradiation (PBI). Postoperative PBI leads to unnecessary large irradiated breast volumes due to artifacts, including seroma.^1^ Preoperative PBI allows more precise target volume definition as the tumor is still in situ, and more importantly carries the possibility of tumor downstaging.

## Present

Recent studies have reported good to excellent clinical and oncological outcomes in patients treated with preoperative PBI followed by BCS.^2^ Five studies reported results on single-dose external beam preoperative PBI ranging between 15 and 21 Gy.^2^ Treatment with this relatively high dose led to mainly mild toxicity and good to excellent cosmetic outcomes in the majority of the patients. A remarkable result is the increased rate of pathologic complete response (pCR) after a longer interval of 6–8 months (42%) in comparison with a shorter interval of 2–8 weeks between radiotherapy and surgery (0–15%).^2^ Therefore, extending the interval could lead to an even higher response rate.

## Future

The ultimate goal is to omit surgery in future low-risk patients with a pCR. For this purpose, it is necessary to be able to predict a pCR accurately after single-dose PBI. Even when patients do not reach a pCR after preoperative PBI, the advantage of this approach is single-dose radiotherapy instead of the standard multi-fractionated radiotherapy (i.e., 5–15 fractions).^3^ This could reduce the treatment burden, facilitate healthcare logistics, and reduce healthcare costs. The positive predictive value of magnetic resonance imaging (MRI) alone is 67%, which is not sufficient to predict a pCR.^4^ Combining MRI parameters with biomarkers, such as gene expression profiling, specific immune profiles, and circulating tumor DNA could result in more accurate prediction of pCR after preoperative single-dose PBI in low-risk breast cancer patients.^5^

## References

[1] Nichols EM, Dhople AA, Mohiuddin MM, Flannery TW, Yu CX, Regine WF (2010). Comparative analysis of the post-lumpectomy target volume versus the use of pre-lumpectomy tumor volume for early-stage breast cancer: implications for the future. Int J Radiat Oncol Biol Phys.

[2] Civil YA, Jonker LW, Groot Koerkamp MPM (2023). Preoperative partial breast irradiation in low-risk breast cancer patients: a systematic review of literature. Ann Surg Oncol.

[3] Murray Brunt A, Haviland JS, Wheatley DA (2020). Hypofractionated breast radiotherapy for 1 week versus 3 weeks (FAST-Forward): 5-year efficacy and late normal tissue effects results from a multicentre, non-inferiority, randomised, phase 3 trial. Lancet.

[4] Vasmel JE, Charaghvandi RK, Houweling AC (2020). Tumor response after neoadjuvant magnetic resonance guided single ablative dose partial breast irradiation. Int J Radiat Oncol Biol Phys.

[5] Papakonstantinou A, Gonzalez NS, Pimentel I (2022). Prognostic value of ctDNA detection in patients with early breast cancer undergoing neoadjuvant therapy: a systematic review and meta-analysis. Cancer Treat Rev.

